# Supplementary material for: Enhanced Non-Invasive Diagnosis of Female Urinary Incontinence Using Static and Functional Transperineal Ultrasonography
Source: Diagnostics (Basel). 2024 Nov 14;14(22):2549. doi: 10.3390/diagnostics14222549 (PMC11592590; doi:10.3390/diagnostics14222549)
Supplement: Supplementary file 1 [file diagnostics-14-02549-s001.zip › diagnostics-3256685-supplementary.pdf]

Supplementary Table S1. Results of logistic regression analyses for the detection of urinary incontinence with two different approaches including adjustment for age, body mass index, or presence of clinical pelvic organ prolapse

	OR	95% CI	p-value for the variable
Model 1: Ultrasound parameters based on anatomical angles and diameters (BSD; alpha, beta, gamma, and retrovesical angles; urethral diameter).			
BSD difference between rest and the Valsalva maneuver (mm)	1.15	1.05-1.27	0.0039
Mean urethral diameter (mm)	4.28	2.07-8.83	0.0001
Model 1 adjusted for age, BMI, and presence of POPQ (p for model < 0.0001)			
BSD difference between rest and the Valsalva maneuver (mm)	1.14	1.03-1.27	0.01
Mean urethral diameter (mm)	4	1.94-8.26	0.0002
Age (years)	0.99	0.93-1.07	0.82
BMI (kg/m^2)	0.96	0.81-1.14	0.65
Presence of POPQ	0.98	0.16-5.9	0.98
Model 2: Presence of specific qualitative signs on ultrasound examinations			
Funneling sign during the Valsalva maneuver	21	6.1-71.9	<0.0001
Model 2 adjusted for age, BMI, and presence of POPQ (p for model = 0.02)			
Funneling sign during the Valsalva maneuver	18.5	4.95-68.8	<0.0001

Age (years)	0.99	0.94-1.05	0.83
BMI (kg/m^2)	0.97	0.86-1.1	0.63
Presence of POPQ	0.79	0.17-3.57	0.76

BMI, body mass index; BSD, bladder–symphysis distance; CI, confidence interval; OR, odds ratio; POPQ, clinical pelvic organ prolapse.

Supplementary Material. Patient-Reported Outcomes and Health History Questionnaire.

PATIENT IDENTIFICATION NUMBER:...

Kraków,……./..…./………..r. (G)

QUESTIONNAIRE

Please fill in the questionnaire below, which is necessary for a proper analysis in the ongoing scientific study. All the information will be kept confidential and will be used only for the purpose of this study.

Name and surname:……………………………………………………………………………(A)

Telephone number:……………………………………………………………………….. (B)

Date of birth:…………………………………………………………………………(C)

Height (cm):..(D)

Weight (kg):...(E)

Maximum weight during your lifetime…………………………...(F)

Please circle the correct answer for each of the following questions:

K. How many times a day do you urinate?

1. <3

2. 3-5

3. 5-10

4. >10

L. How many times during the night do you wake up to go to the toilet to urinate?

1. 0

1. 1

2. 2

3. ≥3

M. Do you often get a “sudden urge to urinate”?

1. No

2. Yes, everyday

3. Yes, sometimes

N. Do you ever feel like your bladder is not fully empty?

1. No

2. Yes, everyday

3. Yes, sometimes

O. Do you ever experience an uncontrollable leakage of urine not related to exercising or lifting?

1. No

2. Yes, once a day

3. Yes, several times a day

3. Yes, but not everyday

P. Do you ever experience an uncontrollable leakage of urine while coughing, sneezing or exercising?

1. No

2. Yes, once a day

3. Yes, several times a day

3. Yes, but not everyday

Q. Do you ever experience an uncontrollable leakage of urine while changing position, e.g. from supine to standing?

1. No

2. Yes, but not every time I change position

3. Yes, every time I change position

R. Do you ever experience any of the following symptoms while urinating?

1. Slow urine flow

2. Intermittent urine flow

3. Split urine stream

S. Do you ever experience "vaginal dryness”?

1. No

2. Yes, everyday

3. Yes, sometimes

T. Do you experience a feeling of lowering of the position of your reproductive organs? (the reproductive organs do not come out of your vaginal opening):

1. No

2. Yes, sometimes

3. Yes, often

4. Yes, always

U. Do you experience a feeling of your reproductive organs (vagina, uterus) falling out? (the reproductive organs come out of your vaginal opening):

1. No

2. Yes, sometimes

3. Yes, often

4. Yes, always

V. Do you ever have to push the reproductive organs inside manually?

1. Yes

2. No

3. Sometimes

W. Does the severity of pelvic organ prolapse depend on the time of the day:

1. No

2. Yes, in the morning

3. Yes, in the afternoon

4. Yes, in the evening

5. Yes, at night

X. Have you ever experienced an uncontrolled passage of stool?

1. No

2. Yes, everyday

3. Yes, sometimes

Y. Have you ever experienced an uncontrolled passage of stool during vaginal intercourse?:

1. No

2. Yes, often

3. Yes, sometimes

Z. Have you ever experienced a sudden need to pass stool?:

1. No

2. Yes, often

3. Yes, sometimes

AA. Have you ever felt an incomplete bowel movement?:

1. No

2. Yes, often

3. Yes, sometimes

AB. How often do you have bowel movements?:

(times / a week)

………………../………………..

AC. Do you have to strain while passing stool?:

1. No

2. Yes, sometimes

3. Yes, often

AD. Have you ever given birth?

1. No

2. Yes

If YES – please complete the table below:

PREGNANCY	Year	Vaginal deliveries (1)<br>Caesarean sections (2)	Newborn's birth weight
1			
2			
3			
4			
5			
6			

AE. If you delivered vaginally, did you have an episiotomy?

1. No

2. Yes

AF. What was your age when the symptoms of pelvic organ prolapse and/or urinary incontinence (stated above) first appeared?

…………………………………………………
………………………………………………….

AG. Have you ever been treated for **constipation**?

1. No

2. Yes

AH. Have you ever been treated for a **chronic cough**?

1. No

2. Yes

AI. Have you ever been diagnosed (by a physician) with any of **the following diseases?**

(Please also state whether the disease or condition has been treated by a physician.)

AJ. **Hypertension?**

1. No

2. Yes

AK. **Diabetes?**

1. No

2. Yes

AL. **Chronic obstructive pulmonary disease (COPD)?**

1. No

2. Yes

AM. Recurrent urinary tract infections (bladder infections) – 3 or more a year?

1. No

2. Yes

AN. Have you ever tried a conservative treatment of pelvic organ prolapse (vaginal pessaries, balls and cones)?

1. No

2. Yes

AO. Have you ever undergone a gynecological **surgery**?

1. No

2. Yes

AP. If yes, what was the procedure?:

1. Abdominal hysterectomy

2. Vaginal hysterectomy

3. Vaginoplasty

4. Other..

....

....

AQ. Have you ever taken or do you still take the following **medications:**

AR. **Diuretics:**

1. No

2. Yes, I used to take it in the past

3. Yes, I'm currently taking it

AS. **Hormone Replacement Therapy (oral, transdermal):**

1. No

2. Yes, I used to take it in the past

3. Yes, I'm currently taking it

AT. **Vaginal estrogens:**

1. No

2. Yes, I used to take it in the past

3. Yes, I'm currently taking it

AU. Do you smoke cigarettes?

1. No, I have never smoked

2. No, but I used to smoke in the past

3. Yes, I smoke <10 cigarettes a day

4. Yes, I smoke >10 cigarettes a day

AV. Have you ever done physical work that involved carrying heavy objects?

1. No, never

2. Yes, in the past I used to carry heavy objects frequently

3. Yes, I still work physically and I often carry heavy objects

AW. Do you exercise on a regular basis?

1. No, never

2. No, but I used to exercise regularly in the past

3. Yes, ≥ 30 minutes at least 3 times a week

4. Yes, ≥ 30 minutes at least once a week

AX. Have you ever done pelvic floor exercises (Kegel exercises)?:

1. No, never

2. Yes, at least once a week

3. Yes, everyday

AY. Are you sexually active?

1. No, but it is not related to my gynecological problems

2. No, I stopped being sexually active when the gynecological problems started

3. Yes

AZ. If yes, do you feel any of the following (please circle the correct answers):

1. Pain during sexual intercourse

2. A feeling of vaginal “looseness” or “widened vagina”

3. Uncontrollable leakage of urine during sexual intercourse

4. Others...

5. I don’t experience any of the symptoms

BA. How would you rate the quality of your life **before** the appearance of the problems with urinary incontinence and/or pelvic organ prolapse on a scale of 1-5 (1-very bad / 5-very good) : ………………

BB. How would you rate the quality of your life **after** the appearance of pelvic organ prolapse on a scale of 1-5 (1-very bad / 5-very good): …………………………….

BC. How would you rate the quality of your life **after** the appearance of urinary incontinence on a scale of 1-5 (1-very bad / 5-very good): …………………………

Thank you for completing the questionnaire.
